# Supplementary figures and images for: Dengue virus infection induces selective expansion of Vγ4 and Vγ6TCR γδ T cells in the small intestine and a cytokine storm driving vascular leakage in mice
Source: PLoS Negl Trop Dis. 2023 Nov 8;17(11):e0011743. doi: 10.1371/journal.pntd.0011743 (PMC10659169; doi:10.1371/journal.pntd.0011743)

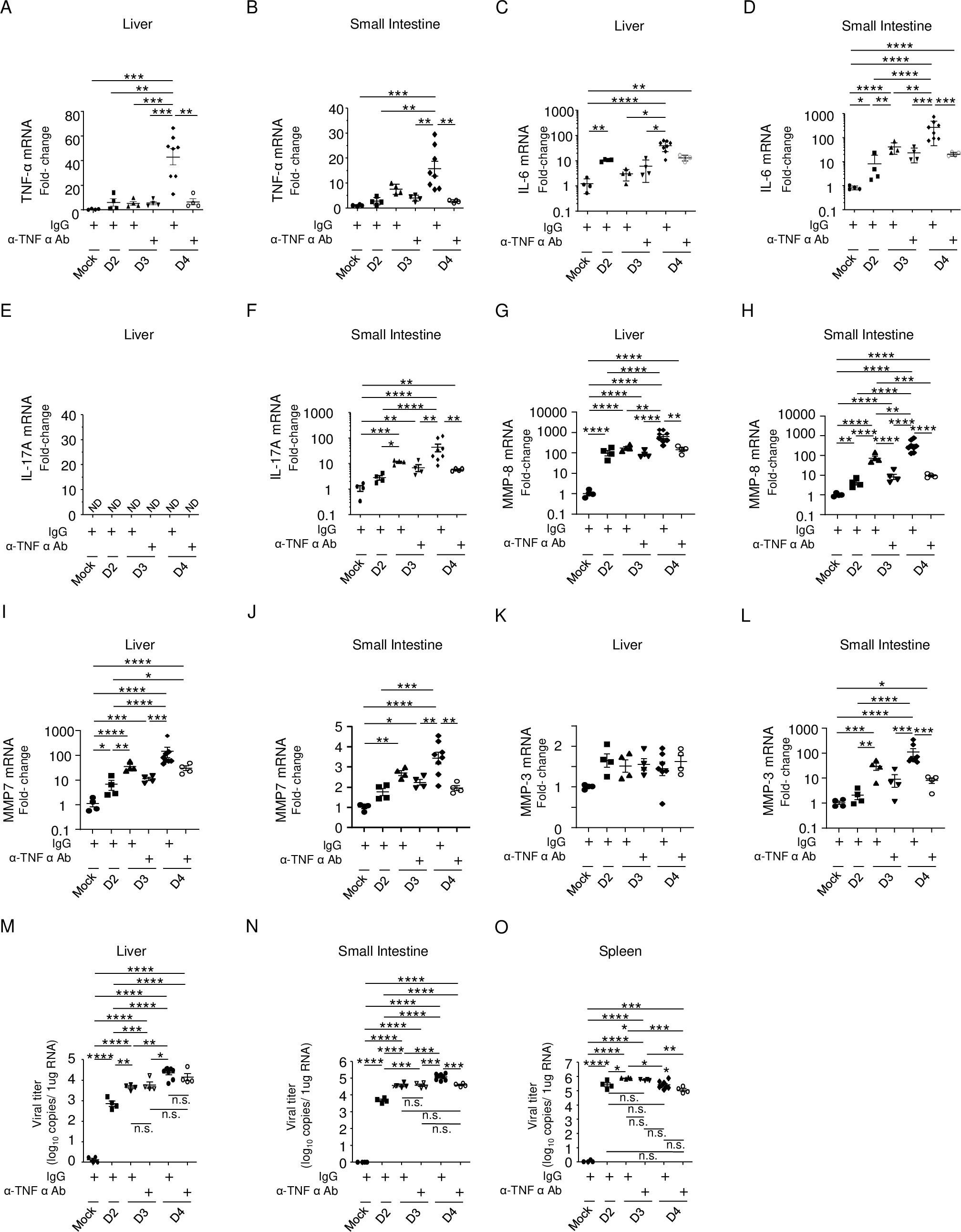

Supplement: S1 Fig — Levels of mRNA expression of TNF-α (A and B), IL-6 (C and D), IL-17A (E and F), MMP-8 (G and H), MMP-7 (I and J), and MMP-3 (K and L) in the liver (A, C, E, G, I, and K) and small intestine (B, D, F, H, J, and L) of mock- (8–10 weeks old) and DENV-3 P12/08 (2 × 10^6^ focus-forming units [FFU])-infected mice. The time points on the x-axis refer to days post-infection. Total RNA was extracted from the liver (n = 4–8) or small intestine (n = 4–8) and subjected to quantitative RT-PCR. The expression of each mRNA was calculated relative to that in mock-infected mice. Viral titers in the liver (M), small intestine (N), and spleen (O) were measured by quantitative RT-PCR. The results are expressed as the mean ± SEM. Each symbol represents an individual mouse. Data were analyzed by one-way ANOVA and significance was assessed by Tukey’s multiple comparison test. *P < 0.05, **P < 0.01, ***P < 0.001, and ****P < 0.0001. (TIF) [file pntd.0011743.s001.tif]

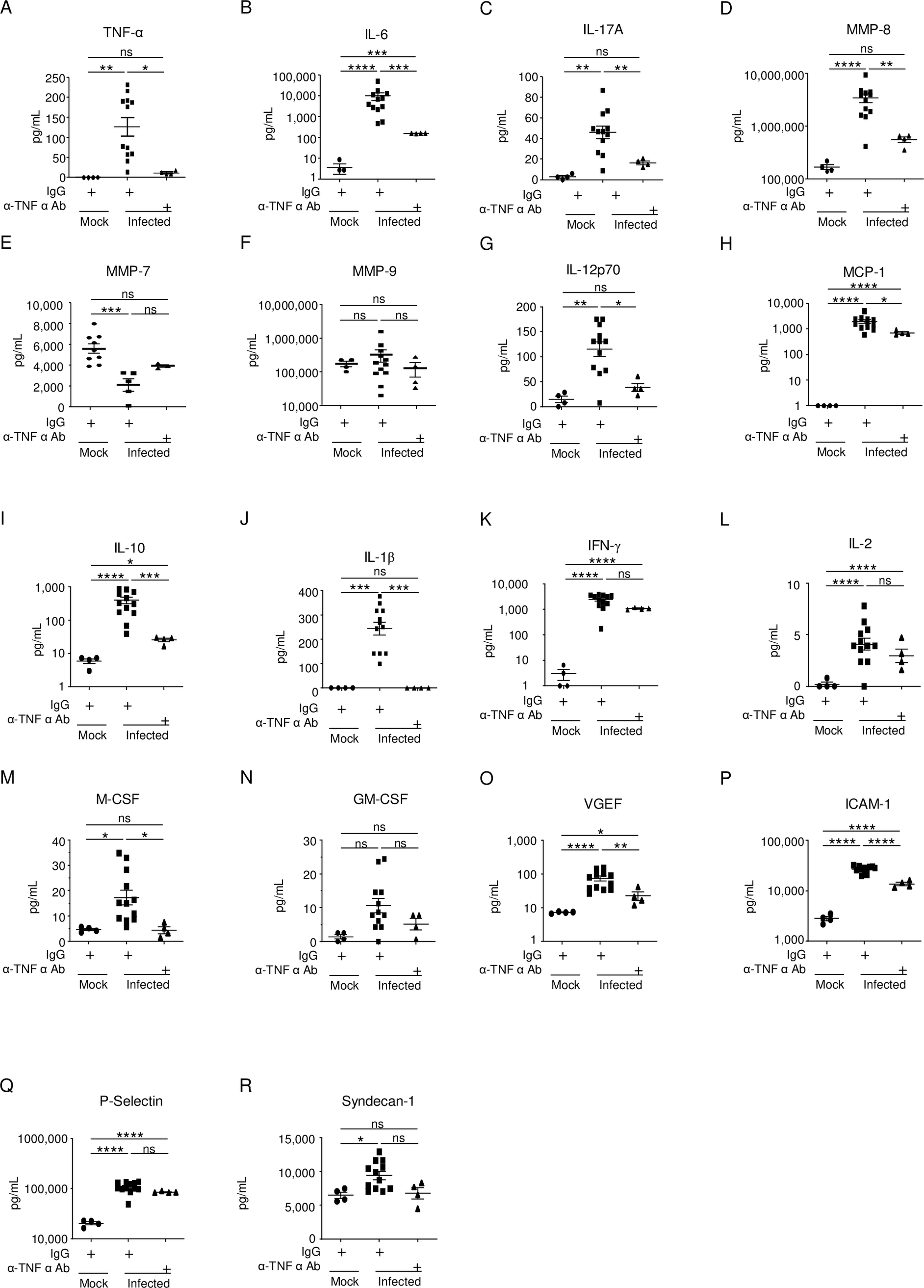

Supplement: S2 Fig — IFN-α/β/γR knockout (KO) mice (8–10 weeks old) were intraperitoneally infected with 2 × 10^6^ focus-forming units (FFU) of DENV-3 P12/08. Isotype control IgG (n = 4), anti-IL-17A Ab (n = 12), or anti-TNF-α Ab (n = 4) was inoculated intraperitoneally at Days 1 and 2 post-infection (p.i.). The mice were sacrificed under anesthesia at Day 4 p.i. and sera were collected. Concentrations of TNF-α (A), IL-6 (B), IL-17A (C), MMP-8 (D), IL-12p70 (E), MCP-1 (F), IL-10 (G), IL-1β (H), IFN-γ (I), IL-2 (J), M-CSF (K), GM-CSF (L), VEGF (M), ICAM-1 (N), P-selectin (O), and syndecan-1 (P) in sera were determined by the Mouse Magnetic Luminex Assay kit. Concentrations of all factors were analyzed by one-way ANOVA. Significance was assessed by Tukey’s multiple comparison test. *P < 0.05, **P < 0.01, ***P < 0.001 and ****P < 0.0001. ns indicates no significant difference. (TIF) [file pntd.0011743.s002.tif]

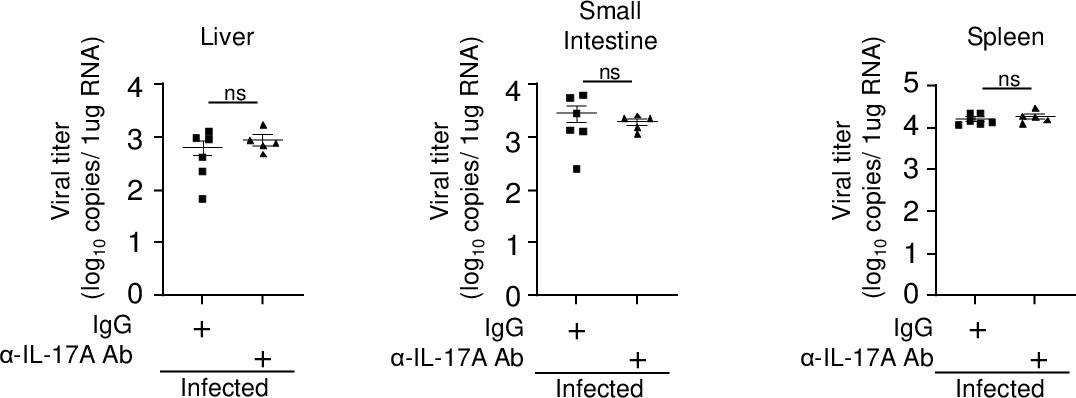

Supplement: S3 Fig — IFN-α/β/γR knockout (KO) mice (8–10 weeks old) were infected with 2.0 × 10^6^ focus-forming units (FFU) of DENV-3 P12/08. Anti-IL-17A Ab (n = 4) or isotype control IgG (n = 6) was injected intraperitoneally at Days 1 and 2 post-infection, and the mice were sacrificed at Day 4 post-infection. Viral copy numbers in the liver, intestine, and serum were determined by quantitative RT-PCR. Differences in viral titers were analyzed by Student’s t-test. ns indicates no significant difference (P > 0.05). The results are expressed as the mean ± SEM. (TIF) [file pntd.0011743.s003.tif]

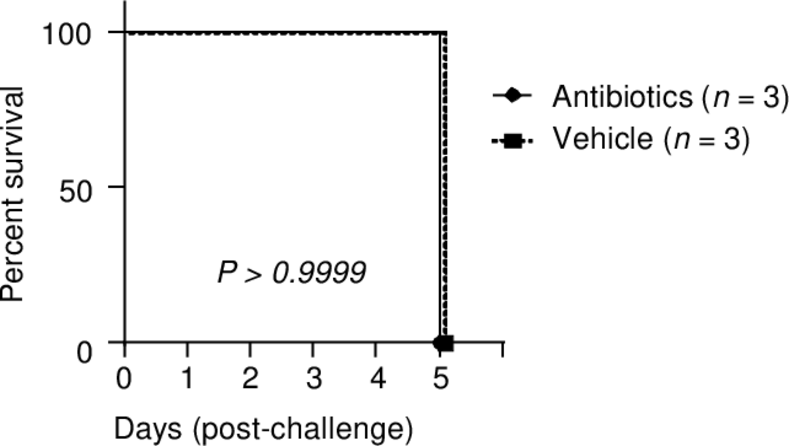

Supplement: S4 Fig — IFN-α/β/γR knockout (KO) mice (8 weeks old) were administered a combination of four antibiotics, consisting of 1 mg/ml ampicillin (Nacalai Tesque), 1 mg/ml neomycin (Nacalai Tesque), 1 mg/ml metronidazole (Nacalai Tesque), and 500 μg/ml vancomycin (Nacalai Tesque) in sterilized drinking water for 2 weeks. IFN-α/β/γRKO mice were intraperitoneally infected with 2 × 10^6^ focus-forming units (FFU) of DENV-3 P12/08 (n = 3/group), and the mice were observed up until Day 5 post-infection. Kaplan–Meier survival curves show the percentage of mice surviving on the specified days post-infection. Significant differences between individual groups were evaluated using the log-rank (Mantel–Cox) test. P > 0.9999. (TIF) [file pntd.0011743.s004.tif]

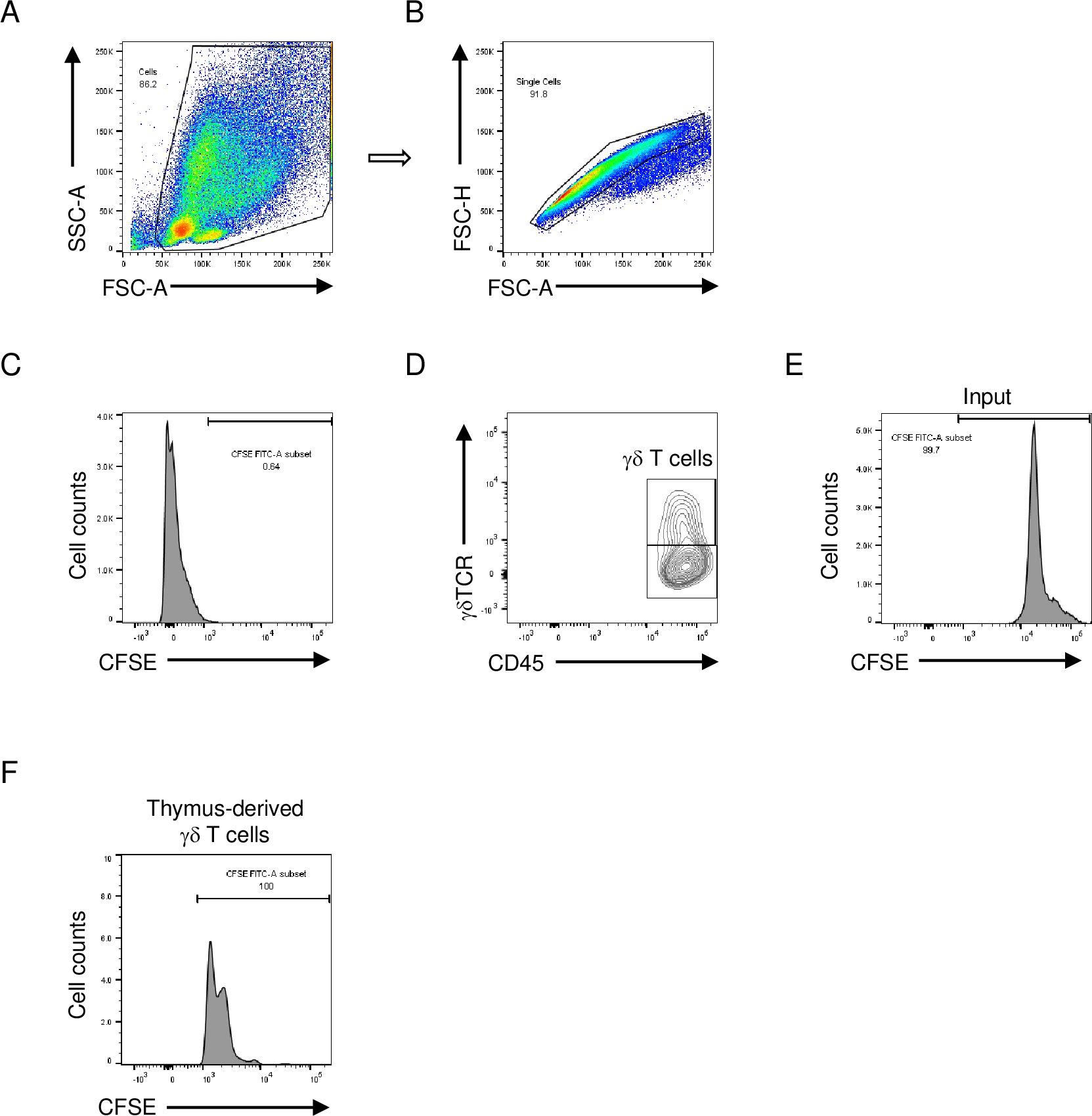

Supplement: S5 Fig — Representative flow cytometry plots of gated intestinal cells. IFN-α/β/γR knockout (KO) mice (8–10 weeks old; n = 3/group) were intraperitoneally infected with 2 × 10^6^ focus-forming units (FFU) of DENV-3 P12/08 or mock. CFSE-labeled cells from the thymus or the intestine were intravenously transferred, and the mice were sacrificed under anesthesia at Day 4 post-infection. The small intestine was collected. (A) The SSC-FSC profile was used to distinguish leukocyte populations from other cell populations. (B) Duplets were removed by singlet gating and debris was removed from the analysis. (C) CFSE-positive cells were further gated. (D) γδ T cells were gated by γδTCR-CD45 analysis. (E) CFSE-labeled input cells. (F) Thymus-derived CFSE-labeled γδ T cells isolated from the small intestine of infected IFN-α/β/γRKO mice. (TIF) [file pntd.0011743.s005.tif]

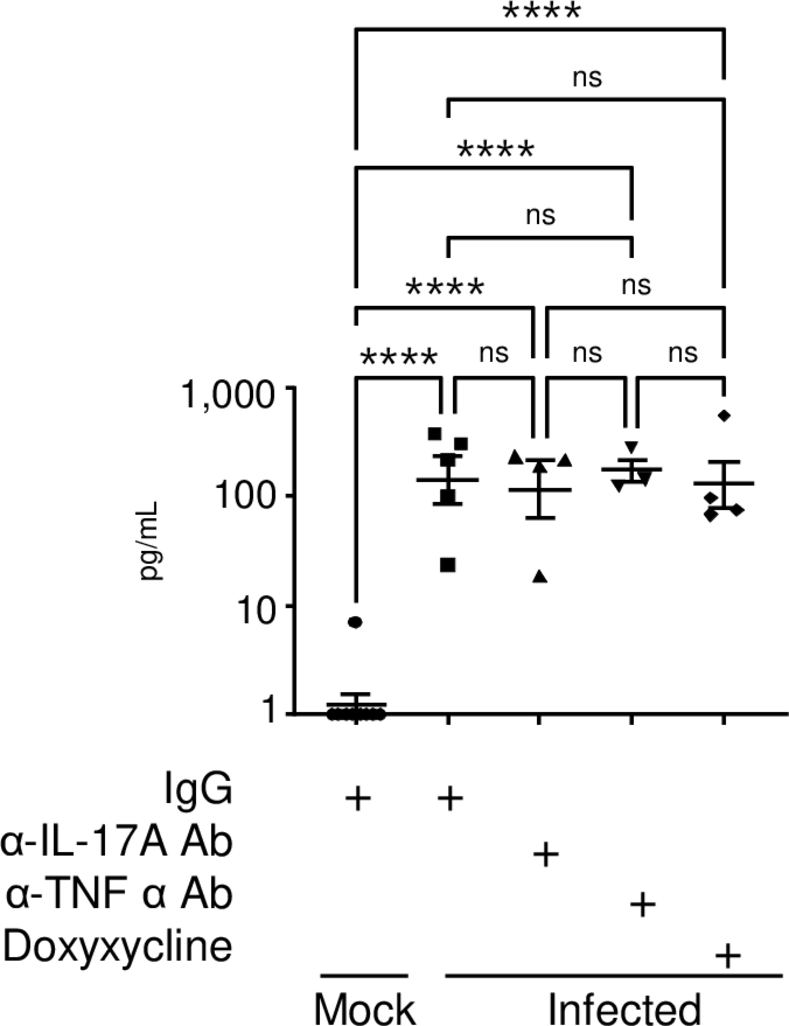

Supplement: S6 Fig — IFN-α/β/γR knockout (KO) mice (8–10 weeks old) were intraperitoneally infected with 2 × 10^6^ focus-forming units (FFU) of DENV-3 P12/08 or mock (n = 9). Isotype control IgG (n = 5), anti-TNF-α (n = 4), or anti-IL-17A Ab (n = 3) was intraperitoneally inoculated at Days 1 and 2 post-infection, and the mice were sacrificed under anesthesia at Day 4 post-infection. Sera were then collected. Serum IL-23 concentrations were determined by the Mouse IL-23 Quantikine ELISA Kit (R&D systems). IL-23 concentrations were analyzed by one-way ANOVA. Significance was assessed using Tukey’s multiple comparison test. *P < 0.05, **P < 0.01, ***P < 0.001, and ****P < 0.0001. ns indicates no significant difference. (TIF) [file pntd.0011743.s006.tif]
