# Supplementary material for: Dengue virus infection induces selective expansion of Vγ4 and Vγ6TCR γδ T cells in the small intestine and a cytokine storm driving vascular leakage in mice
Source: PLoS Negl Trop Dis. 2023 Nov 8;17(11):e0011743. doi: 10.1371/journal.pntd.0011743 (PMC10659169; doi:10.1371/journal.pntd.0011743)
Supplement: S1 Table — (DOCX) [file pntd.0011743.s007.docx]

| S1 Table. Upstream regulator analysis | | | | |
| --- | --- | --- | --- | --- |
| Upstream Regulators | **Day3, IgG**  **IgG,**  **-log(*p*-value)** | **Day 3, α-TNF-αAb**  **α-TNF-α Ab,**  **-log(*p*-value)** | **Day4, IgG**  **IgG,**  **-log(*p*-value)** | **Day 4, α-TNF-αAb**  **α-TNF-α Ab,**  **-log(*p*-value)** |
| *Liver* |  |  |  |  |
| IL17A | 7.93 | 4.48 | 13.92 | 11.21 |
| IL1B | 16.22 | 9.87 | 27.29 | 24.81 |
| TNF | 19.12 | 9.11 | 39.50 | 36.81 |
| IL1 | 7.77 | 5.88 | 15.63 | 15.98 |
| CSF1 | 6.80 | 4.43 | 11.54 | 12.39 |
| IL13 | 8.37 | 6.22 | 18.14 | 20.02 |
| IFNG | 23.84 | 13.43 | 32.93 | 36.41 |
| CSF2 | 18.41 | 5.58 | 20.72 | 20.59 |
| IL6 | 16.36 | 8.49 | 25.95 | 29.80 |
| TNFSF11 | 6.34 | 6.54 | 10.70 | 12.78 |
| IL10 | 11.66 | 7.95 | 20.32 | 26.55 |
| IFNB1 | 7.75 | 10.16 | 12.71 | 15.98 |
| IL12 (complex) | 8.55 | 8.22 | 13.29 | 18.20 |
| IL4 | 11.13 | 7.49 | 19.02 | 30.55 |
| IL15 | 5.39 | 3.93 | 9.85 | 16.80 |
| CSF3 | 10.87 | 5.65 | 11.14 | 15.03 |
| IL21 | 4.97 | 5.27 | 9.37 | 17.99 |
| IL2 | 10.56 | 5.50 | 12.14 | 24.49 |
| CD40LG | 7.98 | 5.01 | 7.28 | 12.08 |
| Interferon alpha | 10.40 | 12.93 | 12.97 | 20.48 |
| *Small Intestine* |  |  |  |  |
| IL17A | 16.91 | 14.10 | 19.21 | 11.48 |
| IL6 | 18.85 | 19.16 | 22.84 | 16.04 |
| IL1A | 14.53 | 11.85 | 18.28 | 11.02 |
| IFNG | 21.64 | 16.49 | 39.48 | 14.62 |
| TNF | 25.12 | 15.65 | 40.29 | 15.91 |
| IL13 | 16.17 | 9.96 | 26.96 | 11.18 |
| Interferon alpha | 8.64 | 10.02 | 23.36 | 8.32 |
| IL1 | 14.55 | 14.21 | 21.76 | 14.07 |
| IFNB1 | 5.07 | 4.80 | 16.19 | 4.65 |
| IL4 | 13.71 | 11.70 | 20.43 | 12.46 |
| IL10 | 15.53 | 14.31 | 25.86 | 14.69 |
| IFNA2 | 3.56 | 4.63 | 16.07 | 3.97 |
| IL1B | 32.89 | 25.75 | 47.69 | 28.83 |
| IL2 | 12.53 | 8.52 | 18.37 | 10.32 |
| Ifn | 5.96 | 10.84 | 18.24 | 8.66 |
| IL21 | 5.60 | 6.02 | 18.82 | 6.51 |
| IL27 | 6.05 | 5.44 | 14.74 | 6.46 |
