# Supplementary material for: Dengue virus infection induces selective expansion of Vγ4 and Vγ6TCR γδ T cells in the small intestine and a cytokine storm driving vascular leakage in mice
Source: PLoS Negl Trop Dis. 2023 Nov 8;17(11):e0011743. doi: 10.1371/journal.pntd.0011743 (PMC10659169; doi:10.1371/journal.pntd.0011743)
Supplement: S2 Table — (DOCX) [file pntd.0011743.s008.docx]

| S2 Table. Top analysis-ready molecules | |
| --- | --- |
| Molecules | **Expression Value** |
| *Expression Fold Change up-regulated in the liver* |  |
| REG3B | 564.771 |
| IGDCC4 | 468.451 |
| SULT1E1 | 437.030 |
| FMO3 | 423.488 |
| E030018B13Rik | 347.305 |
| CHI3L1 | 324.201 |
| CXCL13 | 272.068 |
| Stfa1 (includes others) | 230.012 |
| BMPER | 228.686 |
| LC10A6 | 190.568 |
| *Expression Fold Change down-regulated in the liver* |  |
| Hsd3b4 (includes others) | -4945.018 |
| CA2 | -2909.500 |
| Clec2e/Clec2h | -575.492 |
| Clco1a1 | -486.010 |
| INMT | -425.179 |
| SERPINA6 | -353.643 |
| CYP1A2 | -157.574 |
| ELOVL3 | -127.511 |
| Scd3 | -121.702 |
| CYP26A1 | -115.447 |
| *Expression Fold Change up-regulated in the small intestine* |  |
| S100A8 | 3772.267 |
| Saa3 | 1703.061 |
| Chil3/Chil4 | 1422.898 |
| S100A9 | 1390.855 |
| CLEC4E | 1266.287 |
| Sprr2f | 1166.307 |
| IL6 | 659.209 |
| MMP8 | 567.320 |
| Stfa1 (includes others) | 552.042 |
| LCN2 | 512.828 |
| *Expression Fold Change down-regulated in the small intestine* |  |
| LCT | -9610.605 |
| CYP3A5 | -3974.510 |
| S100G | -3936.385 |
| CYP2B6 | -2997.776 |
| Cyp3a25 (includes others) | -2020.592 |
| 2010106E10Rik | -1272.645 |
| MBL2 | -960.941 |
| MME | -868.796 |
| CYP1A1 | -683.515 |
| CYP4A11 | -673.212 |
