# Supplementary material for: Dengue virus infection induces selective expansion of Vγ4 and Vγ6TCR γδ T cells in the small intestine and a cytokine storm driving vascular leakage in mice
Source: PLoS Negl Trop Dis. 2023 Nov 8;17(11):e0011743. doi: 10.1371/journal.pntd.0011743 (PMC10659169; doi:10.1371/journal.pntd.0011743)
Supplement: S3 Table — (DOCX) [file pntd.0011743.s009.docx]

**S3 Table. Real-time PCR primer and probe sequences**

| Gene | Sequence | |  |
| --- | --- | --- | --- |
| TNF-α | Forward | CATCTTCTCAAAATTCGAGTGACAA | |
|  | Reverse | TGGGAGTAGACAAGGTACAACCC | |
| IL-6 | Forward | GAGGATACCACTCCCAACAGACC | |
|  | Reverse | AAGTGCATCATCGTTGTTCATACA | |
| MMP-8 | Forward | TCTTCCTCCACACACAGCTTG | |
|  | Reverse | CTGCAACCATCGTGGCATTC | |
| MMP-3 | Forward | TTTAAAGGAAATCAGTTCTGGGCTATA | |
|  | Reverse | CGATCTTCTTCACGGTTGCA | |
| IL-17A | Forward | GAGCTTCCCAGATCACAGAG | |
|  | Reverse | AGACTACCTCAACCGTTCCA | |
|  | Probe | 56-FAM/TCCACCGCA/ZEN/ATGAAGACCCTGATAG/3IABkFQ | |
| MMP-7 | Forward | GAACAGAAGAGTGACTCAGACC | |
|  | Reverse | GATGCTCACTTTGACAAGGATG | |
|  | Probe | 56-FAM/ATGGTGAGG/ZEN/ACGCAGGAGTGAAC/3IABkFQ | |
