# Supplementary material for: Dengue virus infection induces selective expansion of Vγ4 and Vγ6TCR γδ T cells in the small intestine and a cytokine storm driving vascular leakage in mice
Source: PLoS Negl Trop Dis. 2023 Nov 8;17(11):e0011743. doi: 10.1371/journal.pntd.0011743 (PMC10659169; doi:10.1371/journal.pntd.0011743)
Supplement: Attachment — Submitted filename: Response_to_ReviewersComments_PNTD-D-23-00821_2.docx [file pntd.0011743.s012.docx]

***Reviewer #1: In this study, Kurosu et al showed that a selective expansion of IL-17A-producing γδ T cells in the small intestine is at least partially responsible for the induction of lethal vascular leak caused by dengue virus (DENV) in IFN-α/β/γR KO mice. The critical role of IL-17A in causing severe vascular leak was demonstrated with the 50% protection of mouse survival by the treatment with anti-IL-17A antibodies accompanied with the reduction of cytokine gene expressions, such as TNF-α, in small intestine. In addition, the authors showed that DENV infection causes the infiltration of neutrophils in small intestine, resulting in a massive production of MMP-8, which is also shown to be responsible for the induction of vascular leak. Thus, this study presented novel mechanisms of vascular leak caused by DENV infection in IFN-α/β/γR KO mice; (1) IL-17A produced by γδ T cells and (2) MMP-8 produced by neutrophils. However, I would request more convincing data/explanations to accept the author’s claims.***

***1. Figure 6 is important data to identify the IL-17A-expressing lymphocytes. The gate was first set on CD45-positive and SSC (A). However, the non-lymphocyte population in infected mice seems totally shifted toward the right side of x-axis and the gate included a lot of non-lymphocyte cells. This arises the possibility that the CD3+γδTCR+ population (B) includes a number of non-specific cells. I understand that the activated lymphocytes may shift their positions to higher SSC intensity, but they should be still distributed within the elongated pattern of lymphocyte population. I urge the authors to re-analyze the data with appropriate gating to obtain more convincing results.***

**>Response**
In panel A, the CD45 positive cell population was increased. It is not shifted of non-lymphocyte cells. We measured cells extracted from the intestine of both mock- and infected mice in the same condition. This was a very repeatable observation. As shown in Fig. 6F, Fig. 8F and Fig. 9F, infection dramatically increased the infiltrations of γδ T cells (34-fold), neutrophils (45-330-fold), and other monocytes-macrophages. These cells are CD45 positive cells. This is why the right panel of figure 6A (infected) contains many CD45 positive cells. Cells from the small intestine are not only composed of blood cells, but also other cells such as endothelial cells, epithelial cells and so on. We first wanted to separate blood cells from non-blood cells, since most IL-17A-producing cells are blood cells by monitoring CD45 expression. The CD45+/SSC^high^ population is mainly neutrophils and monocytes. Granulocytes and monocytes usually have higher SSC. We believe this is the best way to detect IL-17A-producing cells.

***2. The treatment with anti-IL-17A antibodies showed 50% protective efficacy (Fig.5 A). This is unconvincing to me since the treat mice showed similar pattern of weight reduction to untreated mice (Fig.5B) and similar levels of cytokine production to anti-TNF antibody treatment (Fig. 5 E-M), which showed 100% protective efficacy (Fig. 1F) and induced less weight loss (Fig. 1G). How do the authors explain this discrepancy?***

**>Response**

We appreciate referee’s comment. We think it is an important point, but we did not mention about it to avoid confusion for readers. We had the same question why the body weight change was the same as IgG control when we obtained first result, because we knew that anti-TNFα antibody treatment reduced body weight loss of infected mice at day 4 post-infection (Figure 1G). We then repeated this several times but got the same results, similar body weight loss. The difference is probably due to the different site of action of anti-TNF-α treatment and anti-IL-17A treatment. TNF-α signaling seems to affect several signaling pathways more broadly at an earlier stage. In contract, IL-17A presumably functions during late period to enhance the production of IL-6 (Figure 3B). This may be the reason why the body weight loss was not relieved even when mice were treated with anti-IL-17A antibody. This also affect the difference in the percentage of survival between the two treatments. We have added this part in the discussion (lines 396-402).

***Reviewer #2: PNTD-D-23-00821
Takeshi Kurosu and colleagues submitted a manuscript entitled “Dengue virus infection induces selective expansion of Vγ4 and Vγ6TCR γδ T cells in the small intestine and a cytokine storm driving vascular leakage in mice”. In the manuscript, the authors examined the mechanisms and effects of blockade of TNF-α signaling on DENV infected mice during severe dengue outcome. Using a well-established mouse model of severe dengue which display human symptoms including vascular leakage, the authors performed transcriptomic analysis of the liver and small intestine samples collected chronologically in presence/absence of blockade of TNF-α signaling and evaluated the cytokine and effector level events. They found that (i) blockade of TNF-α signaling has a better effect in pro-inflammatory cytokines in small intestine compared to liver, (ii) a critical role of IL-17A produced by γδ T cells in the small intestine during severe dengue, and (iii) detected the presence of neutrophil infiltrate that produced MMP-8 (Matrix Metalloproteinase 8), a collagen cleaving enzyme which is a major player of inflammatory response.
The manuscript addresses a critical question in the field regarding the mechanisms of action following severe dengue. The manuscript uses well-defined approaches to explore the questions and discuss the conclusions. The manuscript is well written, however some concerns detailed below should be addressed by the authors to improve the understanding and the quality of the manuscript.***

Minor comments:
***Figure 1: Panel E needs more details as done for panel A. The symbols are confusing. When is the infection and what antibody has been used?
The authors mentioned that the mice are dying at day 4 in this mouse model, but Fig 1F showed day 5. It is not clear if the mice are dying at day 4 or 5.
Typos in line 962: Mice were instead of Mice. Were***

**>Response**

Thank you for your suggestion. We added detailed information in Figure 1, panel E. In terms of the time of death of the mice, the mice usually start to show severe symptoms at the end of day 4, die very late period on day 4 or die very early period on day 5. If the condition of the mice did not reach the level to be sacrificed on day 4, we continued to observe the mice until next day. As a result, mice were sometimes found to be dead on day 5 when we observed them. In this case, we counted the mice as dead on day 5 and considered the change in body weight to be valid up to day 4. This happens because the disease progresses rapidly in this model.

Regarding line 962, we have corrected it. We appreciate finding a mistake. We have removed a period after “Mice”

***Figure S2: The effects of TNF-α blockade are mentioned as limited, why? Even though in panel F, the protection is 100%. This is not clear. Please, clarify.***

**>Response**

We agree with the referee. The word ”limited” was too strong. We have removed it.

***Typos in line 254-255 and 257: The γδTCR subsets seem mislabeled. i.e CD3+/γδTCR- (including CD8 T cells) in Fig6D instead of CD3+/γδTCR+ (including CD8 T cells). Please, double check, Figure 6C-G***

**>Response**

We apologize for this. There were mislabels. We also changed We have also changed the writing style, i.e. CD3 comes first and γδTCR later.

Figure S5: Add the title of the Y axis.

**>Response**

We have added the title.

***Figure 8: The gating strategy will be better and easy to follow as one panel instead of 5 panels.***

**>Response**

Thank you for your suggestion. We have changed the figure to make it easier for readers to understand. We have also changed the figure legend and main text to match the new figure.

***Figure 9: The resolution of the image is not great and the authors should add annotations and labels to indicate what histopathological changes should be seen and compared between treatments.***

**>Response**

We have added annotations and labels figure 9. A high resolution figure will be submitted for publication. We have also added a slightly more detailed description to make it easier for readers to understand in the main manuscript and legend (lines 315, 319-320, 323-324, 329). We have also changed Figure 9B as it may not be clear to understand. A new panel B shows a slightly wider area so that readers can see the difference more easily.

Moderate comments:

***In all figures, the authors did not specify how many times the experiments were done. Moreover, in Figure 1, only 3 to 4 mice have been used. This looks like a one-time experiment and knowing the variability of the mouse experiments, it is not acceptable to do an experiment only once. Please, clarify and/or add more samples to confirm the results.***

**>Response**

Thank you for your suggestion. We are sorry that we did not describe them. We have added these descriptions in the figure legends. The microarray experiments were performed only once, but three to five mice were used for each group, and statistically analyzed. We also confirmed the result of important factors by quantitative RT-PCR as shown in the figures.

***Figure 4: It is not clear why the authors are not using a better protocol to blockade the IL-6 signaling. The antibody treatment did not effectively suppress IL-6 signaling as measured by serum albumin A levels (Fig 4C). Is it the result of a suboptimal protocol or a bad antibody lot? The use of MR16-1 to suppress IL-6 signaling seems to have better effects in others published manuscripts. The purpose of this figure is not clear if the treatment is not working.
In addition, this mouse model is supposed to succumb to DENV infection at day 4, however this is not the case in panel A. Could the authors clarify why this mouse model is different here?***

**>Response:**

We had also tested another anti-IL-6 neutralizing MP5-20F3 antibody against the IL-6 ligand, but it did not protect mice well (Phanthanawiboon et al. PLoS One 2016). The MR16-1 is thought to be a better neutralizing antibody to block the IL-6 signaling pathway by blocking a receptor for IL-6, and which is an original mouse clone of the humanized anti-IL-6 antibody tocilizumab, which is widely used to treat autoimmune diseases, such as rheumatoid arthritis, Castleman disease, and so on. The purpose of showing this figure is to convey a message; ”Although IL-6 is a key molecule in severe disease, it may be sometimes difficult to target for therapy. This is due to the high levels of IL-6 during acute disease progression in this mouse model (over 10,000 pg/mL in serum of infected mice in this model). In this case (very high IL-6 level), targeting another regulator molecule, such as IL-17A, for IL-6 may be more effective. Compared to IL-6, concentrations of TNF-α or IL-17A are lower as shown in Figure 5E and 5G. Targeting TNF-α or IL-17A may be more effective. We thought this was important information. On the other hand, we also think that the very high level of IL-6 in the current model may be because our mouse model progresses the disease too severely and too rapidly. In patients with Castleman’s disease or rheumatoid arthritis, IL-6 levels in the blood are usually less than 50-100 pg/mL (Tsukuda et al., PLOS One, 2020; Strand et al., Arthritis Research & Therapy, 2020; Sharma et al, Interleukins, 2021). This is why therapeutic antibodies against IL-6 are effective in these diseases. In cause of human diseases with acute infections such as dengue fever or SARS, the levels of IL-6 levels seem not to be as high as that in this mouse model (Dayarathna et al., Scientific Reports, 2020). In fact, there are many reports suggesting that tocilizumab has reduced mortality in severe COVID-19. We would like to suggest a more detailed and chronological study of the cytokine profiles of dengue patients for immunotherapy. We have included this in the discussion (lines 402-412).

Regarding the day of mouse death in Figure 5, the reviewer is correct. Some mice still survive after 5 days p.i. This may also be slightly influenced by the virus batch we used. When we performed the mouse challenge experiment, we infected several mouse groups with virus from same single tube to avoid artifact. The experiment in Figure 4A-C was performed simultaneously using the same virus source. We believe that the conclusion of “not significant” in figure 4 is correct.

We also added S3 Fig. Then, the S3, S4 and S5 Fig were renamed as S4, S5 and S6 Fig in this submission, respectively.
