# Supplementary material for: Dengue virus infection induces selective expansion of Vγ4 and Vγ6TCR γδ T cells in the small intestine and a cytokine storm driving vascular leakage in mice
Source: PLoS Negl Trop Dis. 2023 Nov 8;17(11):e0011743. doi: 10.1371/journal.pntd.0011743 (PMC10659169; doi:10.1371/journal.pntd.0011743)
Supplement: Attachment — Submitted filename: Response to comments.docx [file pntd.0011743.s013.docx]

***Reviewer #1: Kurosu et al responded to 2 concerns raised by this Reviewer. Overall, their responses are reasonable. One point they missed to answer regarding the FCM data is the possibility that CD3+γδTCR+ population (Fig. 6B) contains a number of non-specific cells by gating a broad range of SSC intensity (Fig. 6A) and this leads the false positive of IL-17A expression (Fig. 6C). I understand that CD45+/SSClow population is mainly lymphocytes and CD45+/SSChigh population would be mainly neutrophils and monocytes. Gating and analyzing separately these populations would be the best way in order to avoid the concern about false-positive and clearly show that γδ T cells are responsible for IL-17A production in small intestine. It would be very easy to address this point.***
**>Response**

Thank you for your suggestion. We think that this is the better presentation for this work because we wanted to include all possible populations. In addition, figure 6H clearly suggests that infection induces proliferation and infiltration of Vγ4, Vγ6TCR γδ T cells, which are well-known IL-17A producers.

***Reviewer #2: The authors addressed some of the comments and improved the manuscript. Typos and grammatical mistakes are still in the manuscript, ie L1065, L1150, etc...*
>Response**

We apologize these mistakes. We corrected them and checked whole manuscript. We had asked a proofreading company to check them. The parts we changed were with blue color highlight.
